# Ninth Version of the AJCC and UICC Nasopharyngeal Cancer TNM Staging Classification

**DOI:** 10.1001/jamaoncol.2024.4354

**Published:** 2024-10-10

**Authors:** Jian-Ji Pan, Hai-Qiang Mai, Wai Tong Ng, Chao-Su Hu, Jin-Gao Li, Xiao-Zhong Chen, James C. H. Chow, Edwin Wong, Victor Lee, Ling-Yu Ma, Qiao-Juan Guo, Qin Liu, Li-Zhi Liu, Ting-Ting Xu, Xiao-Chang Gong, Meng-Yun Qiang, Kwok-Hung Au, Tsz-Chim Liu, Chi Leung Chiang, You-Ping Xiao, Shao-Jun Lin, Yun-Bin Chen, Shan-Shan Guo, Charlene H. L. Wong, Lin-Quan Tang, Zhi-Yuan Xu, Yi-Zhen Jia, Wen-Sa Peng, Li-Ping Hu, Tian-Zhu Lu, Feng Jiang, Cai-Neng Cao, Wei Xu, Jun Ma, Pierre Blanchard, Michelle Williams, Christine M. Glastonbury, Ann D. King, Snehal G. Patel, Raja R. Seethala, A. Dimitrios Colevas, Dai-Ming Fan, Melvin L. K. Chua, Shao Hui Huang, Brian O’Sullivan, William Lydiatt, Anne W. M. Lee

**Affiliations:** 1 Clinical Oncology School of Fujian Medical University, Fujian Cancer Hospital, Fujian Key Laboratory of Translational Cancer Medicine, Fuzhou, China; 2 Xiamen Humanity Hospital, Fujian Medical University Fujian, China; 3 American Joint Committee on Cancer Expert Panel; 4 State Key Laboratory of Oncology in South China, Guangdong Key Laboratory of Nasopharyngeal Carcinoma Diagnosis and Therapy, Guangdong Provincial Clinical Research Center for Cancer, Sun Yat-sen University Cancer Center, Guangzhou, China; 5 The University of Hong Kong–Shenzhen Hospital, Shenzhen, China; 6 Fudan University Shanghai Cancer Center, Shanghai, China; 7 Jiangxi Cancer Hospital and NHC Key Laboratory of Personalized Diagnosis and Treatment of Nasopharyngeal Carcinoma (Jiangxi Cancer Hospital of Nanchang Medical College), Jiangxi, China; 8 Zhejiang Cancer Hospital, Zhejiang, China; 9 Queen Elizabeth Hospital, Hong Kong, China; 10 Pamela Youde Nethersole Eastern Hospital, Hong Kong, China; 11 The University of Hong Kong, China; 12 Princess Margaret Cancer Centre, Toronto, Ontario, Canada; 13 Union for International Cancer Control Head and Neck Core Group; 14 Department of Radiation Oncology, Université Paris-Saclay, INSERM U1018 Oncostat, Gustave-Roussy, Villejuif, France; 15 The Chinese University of Hong Kong, China; 16 American Joint Committee on Cancer Head and Neck Core Group; 17 Memorial Sloan Kettering Cancer Center, New York, New York; 18 University of Pittsburgh Medical Center, Pittsburgh, Pennsylvania; 19 Stanford University School of Medicine, Stanford, California; 20 China Anti-Cancer Association; 21 National Cancer Centre Singapore and Duke-NUS Medical School, Singapore; 22 Nebraska Methodist Health System, Omaha, Nebraska

## Abstract

**Importance:**

Accurate staging is a fundamental step in treating patients with nasopharyngeal carcinoma (NPC) worldwide; this is crucial not only for prognostication, but also for guiding treatment decisions. The American Joint Committee on Cancer (AJCC)/Union for International Cancer Control (UICC) tumor-node-metastasis (TNM) system is the global language for clinicians, researchers, and cancer registries. Continual improvement that aligns with contemporary pattern of care is essential.

**Objective:**

To improve the prognostic accuracy and clinical applicability of the eighth edition (TNM-8) for NPC.

**Design, Setting, and Participants:**

This multicenter study analyzed patients with NPC with detailed tumor features during January 2014 and December 2015 and was reviewed by experienced radiologists. The data analysis was completed in December 2023. The findings were further confirmed with internal and external validation. Statistical analyses and clinical considerations were reviewed by the AJCC/UICC multidisciplinary head and neck panels and attained consensus. The recommendations were evaluated by the AJCC Evidence-Based Medicine Committee before final endorsement as the ninth version (TNM-9).

**Main Outcomes and Measures:**

The primary end point was overall survival. Adjusted hazard ratios of different subgroups were then assessed for confirmation of optimal stage grouping.

**Results:**

Of the 4914 patients analyzed, 1264 (25.7%) were female and 3650 (74.3%) were male; the median (SD) age was 48.1 (12.0) years. Advanced radiological extranodal extension (with involvement of adjacent muscles, skin, and/or neurovascular bundles) was identified as an independent adverse factor for all end points: this was added as a criterion for N3. Patients with nonmetastatic disease were regrouped into stages I to III instead of TNM-8 stages I to IVA. Significant hazard discrimination was achieved by grouping T1-2N0-1 as stage I, T3/N2 as stage II, and T4/N3 as stage III. Although the T1-2N0-1 subgroups had comparable 5-year overall survival, subdivisions into IA (T1-T2N0) and IB (T1-T2N1) were recommended due to the distinction in adjusted hazard ratios following adjustment for chemotherapy use. Metastatic disease was exclusively classified as stage IV, and prognostication was further refined by subdivision into IVA (M1a, ≤3 lesions) and IVB (M1b, >3 lesions). TNM-9 demonstrated superiority compared with TNM-8 in major statistical aspects.

**Conclusion and Relevance:**

The results of this diagnostic study suggest that the ninth version of TNM staging for NPC, based on robust analyses and a comprehensive review by the AJCC/UICC staging committees, provides an improved staging system for global application and a framework for future incorporation of nonanatomical factors. This will be launched for global application in January 2025.

## Key Points

**Question:**

How was the ninth version of the American Joint Committee on Cancer (AJCC) and Union for International Cancer Control (UICC) tumor-node-metastasis (TNM) staging system for improving prognostication of nasopharyngeal carcinoma formulated?

**Findings:**

In this diagnostic study of 4914 patients with nasopharyngeal carcinoma, it was found that substantial improvement could be achieved by adding advanced extranodal extension as a criterion for N3; expanding stage I to include T1-2N0-1 subgroups; subdividing stage I into IA (T1-T2N0) and IB (T1-T2N1); revising stage III (T3/N2M0) and stage IVA (T4/N3M0) to stage II and stage III, confining stage IV to patients with metastatic disease; and subdividing into IVA and IVB (≤3 vs >3 metastatic lesions).

**Meaning:**

This final approved classification will be launched by AJCC and UICC for global application in 2025 and will provide a framework for clinicians, cancer registries, and researchers on clinical trials and further refinement for personalized risk stratification.

## Teaser

This diagnostic study examines the formation of the ninth version of the American Joint Committee on Cancer (AJCC)/Union for International Cancer Control (UICC) tumor-node-metastasis (TNM) staging system.

## Introduction

Accurate prognostication is fundamental for cancer care. Tumor-node-metastasis (TNM) staging based on anatomical factors is a robust prognostic system that is globally applicable. The collaboration between the American Joint Committee on Cancer (AJCC) and the Union for International Cancer Control (UICC) in 1987 laid the foundation for developing a unified system.

Nasopharyngeal carcinoma (NPC) has a skewed geographic distribution, with 83% of new cases originating from Asia,^1^ and exhibits distinct characteristics that require specific therapeutic considerations. The eighth edition of the staging manual (TNM-8), which was introduced in 2017, was a milestone that unified the AJCC/UICC and the Chinese staging system. With substantial improvement in treatment and outcomes, it is essential to reevaluate the prognostic accuracy of the TNM-8 and explore for further potential refinement by incorporating nonanatomical prognostic factors, particularly plasma Epstein Barr virus (EBV)–DNA levels.^2,3,4,5^

A working group consisting of investigators from mainland China and Hong Kong, together with leading representatives from AJCC/UICC staging committees, was established to develop the ninth version of the AJCC/UICC (TNM-9). First, we conducted a comprehensive systematic review of publications on anatomical criteria and nonanatomical prognostic factors for NPC.^6^ Second, we conducted a multicenter validation study to evaluate the published suggestions and identify additional factors (focusing on anatomical parameters in this article). Third, an author (J.M.) was invited to provide external validation^7^; only findings that concurred in both studies were considered. Fourth, the proposed changes were reviewed by the AJCC and UICC International Panel (comprising different disciplines of oncologists, radiologists, and pathologists) for comprehensive assessment of statistical evidence, treatment relevance, and clinical applicability. Only suggestions that attained strong consensus among the panel members were recommended. Fifth, the recommendations were evaluated by the AJCC Evidence-Based Medicine Committee before final endorsement.

## Methods

### Patients

This multicenter study analyzed consecutive patients with histologically confirmed NPC who were treated during 2014 and 2015 in 8 participating centers (with respective ethics approval). All patients with nonmetastatic disease (M0 cohort) had undergone magnetic resonance imaging and a systemic work-up, and all were staged using the TNM-8 system. Detailed radiological features of involved anatomical structures and nodal and distant metastases were reviewed by designated radiologists in each participating center. All M0 patients were treated with curative intent using intensity-modulated (or equivalent) radiotherapy ± chemotherapy as per the current standard of care.^8,9^ The data collection and analyses were approved by the ethical committee of each respective participating center. Informed consent was waived due to the nature of this retrospective clinical study, anonymous measures for ensuring participants' privacy and adhering to data protection regulations, and no involvement of direct intervention or new medical procedures, posing minimal risk to participants. This study followed the Strengthening the Reporting of Observational Studies in Epidemiology (STROBE) reporting guidelines.

### Statistical Analysis

The primary end point was overall survival (OS); secondary end points included local, regional nodal, and distant failure-free survival (L-FFS, N-FFS, and D-FFS, respectively). For the analyses of stage grouping in the M0 cohort, patients within each participating center were randomly assigned to the training set or the validation set in a 7:3 ratio. Further bootstrap testing with 5000 replicates was conducted to ensure robust internal validation. Recursive-partitioning analysis using the STREE program^10^ was performed to provide the preliminary framework; adjusted hazard ratios (aHRs) of different subgroups were then assessed for confirmation of optimal stage grouping. Finally, comparison of TNM-9 with TNM-8 in different statistical aspects was performed. All statistical analyses were conducted with R, version 4.2.2 (R Foundation), and a 2-sided *P* < .05 was considered statistically significant.

## Results

### Overall Cohort and Treatment Characteristics

Of the 4914 patients analyzed, 1264 (25.7%) were female and 3650 (74.3%) were male; the median (SD) age was 48.1 (12.0) years. eTable 1 in Supplement 1 summarizes the basic characteristics of the whole series (N = 4914 patients): 4794 of 4914 patients (97.5%) had nonkeratinizing carcinoma. The median (IQR) follow-up duration was 68 (48-78) months.

In the M0 cohort (n = 4701), 530 of 4701 patients (11.3%) were treated with radiotherapy alone, while the remaining 4171 (88.7%) received combined modality treatment using different chemotherapy regimens and sequences. Among the M1 cohort (n = 213), 96 of 213 (45.1%) underwent investigation with positron emission computed tomography–computed tomograph (PET-CT); 171 of 213 (80.3%) received locoregional radiotherapy for the primary tumor and 103 of 213 (48.4%) received local treatment (surgery and/or radiotherapy) for metastatic lesions in addition to systemic therapy.

Among the host and treatment factors, age (hazard ratio [HR], 1.05 [95% CI, 1.04-1.06] per year increase), sex (male compared with female: HR, 1.40; 95% CI, 1.15-1.69), and addition of chemotherapy (yes vs no: HR, 0.56; 95% CI, 0.45-0.70) were significant factors for OS on univariable analyses (UVA). Therefore, besides the basic set of multivariable analyses (MVA) that were adjusted for age and sex, we had performed an additional set with adjustment for age, sex, and chemotherapy to evaluate the significance independently of treatment effects.

### T Category

eTable 2 in Supplement 1 summarizes the significance of each anatomical structure within the respective TNM-8 T categories. For the TNM-8 T2 category, only 4 of 818 patients (0.5%) had lateral pterygoid muscle involvement; UVA showed significantly worse OS, but MVA was not applicable due to the small sample size. Medial ± lateral pterygoid muscle(s) involvement was identified in 83 of 818 patients (10.1%), and their OS was significantly worse than the remaining T2 (aHR, 2.52; 95% CI, 1.48-4.26) and comparable with T3 (aHR, 1.21; 95% CI, 0.92-1.58). However, upclassifying to T3 was not recommended by the AJCC and UICC panel (only 3 of 12 agreed), given the few patients affected, lack of support by the independent external study,^7^ and conflicting results in the literature.^11,12,13^

For the TNM-8 T3 category, none of the individual structures showed significant differences in unadjusted HRs for OS compared with the overall T3 groups. The data did not support upclassifying paranasal sinus, as suggested by other studies,^13,14,15^ or down-classifying early skull base invasion (including medial ± lateral pterygoid plate, pterygomaxillary fissure, pterygopalatine fossa, and/or floor of sphenoid sinus) as suggested by the external validation study.^7^

For the TNM-8 T4 category, patients with inferior orbital fissure involvement (n = 104) had a significantly worse OS compared with those without. However, further subdivision and upclassifying to a new T4b category was not recommended since this had minimal clinical implications.

Comparisons of aHRs between the respective T categories showed no significant differences for TNM-8 T2 vs T1 for all end points and no significant differences for T3 vs T2 for OS and D-FFS. Although merging T1 and T2 may improve the distinction for OS (aHR, 1.31; 95% CI, 1.05-1.63) and L-FFS (aHR, 1.81; 95% CI, 1.32-2.49), this was not associated with improvement for the distinction for D-FFS (aHR, 1.34; 95% CI, 0.99-1.54). Therefore, no changes were proposed for the T category classification.

### N Category

Detailed analyses of different nodal features (Table 1) showed that the presence of advanced extranodal extension (ENE) (unequivocal involvement of adjacent muscle, skin, and/or neurovascular structures) was an independent adverse prognostic factor for all end points (aHR for OS, 1.67; 95% CI, 1.26-2.19). Thus, in addition to the current criteria of lower neck extension (below the caudal border of the cricoid cartilage) and maximum dimension (>6 cm), addition of advanced ENE as a criterion for N3 was proposed. This suggestion was supported by the external validation study^7^ and other recent publications^16,17,18,19,20^; consensus by the AJCC and UICC panel was 100% (12 of 12).

**Table 1.** Significance of Different Nodal Features Among 4150 Patients With Nonmetastatic Node-Positive Disease^a^

Nodal features	Unadjusted		Adjusted			
			Without chemotherapy		With chemotherapy	
	HR (95% CI)	*P* value	HR (95% CI)	*P* value	HR (95% CI)	*P* value
**OS**						
Maximum dimension (>6 vs ≤6 cm)	2.69 (1.94-3.74)	<.001	1.67 (1.12-2.49)	.01	1.73 (1.16-2.58)	.01
Nodal necrosis (yes vs no)	1.24 (1.04-1.48)	.01	1.17 (0.95-1.44)	.14	1.20 (0.97-1.48)	.09
Laterality (bilateral vs unilateral)	1.49 (1.23-1.80)	<.001	1.23 (0.98-1.54)	.08	1.23 (0.98-1.55)	.07
Extension (below vs above caudal border of cricoid cartilage)	1.93 (1.60-2.34)	<.001	1.54 (1.21-1.97)	<.001	1.50 (1.17-1.92)	.001
Extranodal extension (advanced vs no)^b^	2.42 (1.88-3.11)	<.001	1.67 (1.26-2.19)	<.001	1.67 (1.26-2.20)	<.001
**D-FFS**						
Maximum dimension (>6 vs ≤6 cm)	2.16 (1.49-3.12)	<.001	1.04 (0.68-1.59)	.85	1.05 (0.69-1.60)	.81
Nodal necrosis (yes vs no)	1.52 (1.27-1.83)	<.001	1.34 (1.09-1.66)	.01	1.36 (1.10-1.68)	.004
Laterality (bilateral vs unilateral)	1.53 (1.25-1.87)	<.001	1.30 (1.03-1.65)	.03	1.31 (1.03-1.65)	.03
Extension (below vs above caudal border of cricoid cartilage)	2.67 (2.21-3.22)	<.001	2.14 (1.71-2.68)	<.001	2.14 (1.71-2.68)	<.001
Extranodal extension (advanced vs no)^b^	3.03 (2.40-3.83)	<.001	2.14 (1.66-2.76)	<.001	2.14 (1.66-2.75)	<.001
**N-FFS**						
Maximum dimension (>6 vs ≤6 cm)	1.57 (0.74-3.36)	.24	0.59 (0.21-1.65)	.31	0.59 (0.21-1.65)	.31
Nodal necrosis (yes vs no)	1.69 (1.22-2.33)	.002	1.23 (0.85-1.79)	.27	1.23 (0.85-1.79)	.27
Laterality (bilateral vs unilateral)	1.32 (0.93-1.87)	.12	1.33 (0.88-2.01)	.18	1.33 (0.88-2.00)	.18
Extension (below vs above caudal border of cricoid cartilage)	1.79 (1.24-2.58)	.002	1.57 (1.03-2.38)	.04	1.57 (1.03-2.38)	.04
Extranodal extension (advanced vs no)^b^	2.53 (1.61-3.96)	<.001	2.03 (1.26-3.28)	.004	2.03 (1.26-3.28)	.004

Abbreviations: D-FFS, distant failure-free survival; HR, hazard ratio; N-FFS, nodal failure-free survival; OS, overall survival.

^a^ Multivariable analyses: covariables included age, sex, T category, and significant nodal features ± chemotherapy.

^b^ Extranodal extension–advanced (unequivocal involvement of adjacent muscle skin and/or neurovascular structures).

While advanced ENE was a strongly significant factor, limited ENE to adjacent fat (0.87; 95% CI, 0.63-1.20) or coalescent nodes (1.40; 95% CI, 0.89-2.22) was not significant. Analyses of other nodal features, including parotid node and nodal necrosis, were also not significant for further consideration. Comparisons of aHR between the respective TNM-8 N categories showed that the differences between N1 vs N0 were insignificant for OS and N-FFS, but N1 patients had significantly worse D-FFS compared with N0 patients such that merging of N0 and N1 was not recommended.

### Stage Grouping for the M0 Cohort

Based on the new N classification, we proceeded to assess prognostication according to respective TNM stage groups in the M0 cohort (n = 3205). Using a recursive-partitioning analysis, M0 patients were categorized into 3 prognostic groups (eFigure 1 in Supplement 1): the worst group consisting of T4 (any N) and T1-3N3; the intermediate group consisting of T3N0-2; and the best group consisting of T1-2N0-1 and T1-2N2. Evaluation by aHRs agreed with this preliminary framework, except for further improvement by changing T1-2N2 to the intermediate group. The aHR adjusted for age and sex (without chemotherapy) was 1 to 2.5 or less for stage I (inclusive of T1-2N0-1), 2.5 to 5.0 or less for stage II (inclusive of T1-2N2 and T3N0-2), and more than 5.0 for stage III (inclusive of any T4 or N3). Analyses with additional adjustment for chemotherapy concurred with this fundamental categorization (the corresponding AHR cut-off values changed to 1 to ≤3.5, >3.5 to ≤7.5, and >7.5 for stages I-III, respectively).

The expansion of stage I to include T1-2N0-1 was indicated, as the 5-year OS rates for these subgroups ranged narrowly from 94.8% to 98.4% (eFigure 2 in Supplement 1). Following this change, the TNM-8 stage III and IVA will be revised to TNM-9 stages II and III, respectively. This regrouping of all M0 patients concurred with the external validation study^7^ and other publications.^21,22^ This resulted in significant hazard distinction between adjacent stages (eTable 3 in Supplement 1) and was supported by 11 of 12 members (94% ) of the AJCC and UICC panel.

Detailed analyses of treatment showed marked variation among these 4 early subgroups: a significantly lower proportion of N1 patients were treated with radiotherapy alone compared with N0 (16% vs 64%; *P* < .001). Furthermore, MVA with the inclusion of chemotherapy revealed that the N1 subgroup fared significantly worse (2.84; 95% CI, 1.05-7.71; *P* = .04) than the N0 subgroup. Therefore, 9 of 11 members (91%) of the AJCC and UICC panel recommended subdivision of stage I into IA (T1-2N0) and IB (T1-2N1) for treatment consideration. This was supported by the external validation study.^7^ Repeating the analyses in the internal validation set (n = 1496) that was comparable with the training dataset in terms of host factors and stage distribution showed similar results (eTables 3 and 4 in Supplement 1).

The TNM-9 outperformed TNM-8 in different statistical aspects, including hazard discrimination, hazard consistency, C-index, likelihood difference, Brier score, and distribution. The superiority of overall ranking by TNM-9 vs TNM-8 was demonstrated in 4595 of 5000 (92%) of the bootstrap replicates (eTable 5 in Supplement 1).

### M Category for the M1 Cohort

Analyses of different metastatic features in the M1 cohort (n = 213) showed that the number of metastatic lesions and number of involved anatomic sites/organs were significant prognostic factors on UVA (eTable 6 in Supplement 1). For the 151 patients with metastases to a single organ, those with liver and distant lymph node(s) had higher but nonsignificant unadjusted HRs for OS compared with lung metastases. The number of metastatic lesions was the only independent prognostic factor on MVA. Subdivision of M1 into M1a (≤3 lesions) and M1b (>3 lesions) and a corresponding subdivision of TNM-9 stage IV into IVA and IVB was associated with a significant distinction of OS between the 2 subgroups (60.7% vs 44.2% at 5 years; *P* = .01).

The external validation study^7^ confirmed that the number of metastatic lesions was a significant factor, and the optimal cut-off was 3 or fewer vs more than 3 lesions, but it also suggested to include liver involvement as an additional criterion.^7^ Detailed review of their data by the AJCC and UICC panel showed that, although the addition of the liver criterion could widen the distinction between subgroups (especially for patients who underwent PET-CT), the overall additional benefit was marginal, as only a small subset of M1 patients (35 of 213 [16%]) had 3 or fewer lesions with liver involvement. Thus, all panel members (12 of 12) agreed to adopt 3 or fewer vs more than 3 lesions as the criterion for subdividing TNM-9 M1 and the corresponding stage IV.

### Distribution and Prognostication for the Entire Series

**Figure 1.**
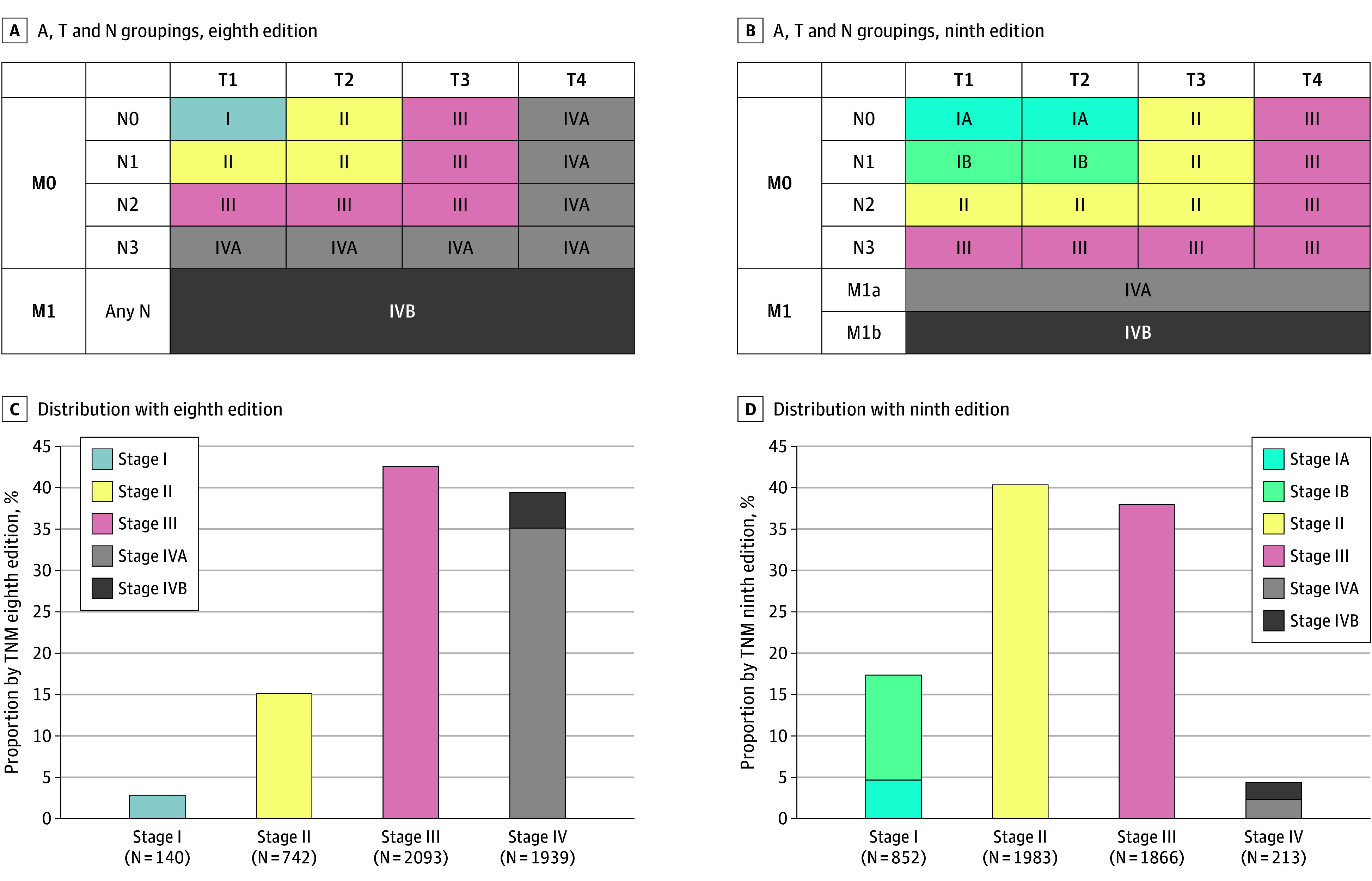
Tumor-Node-Metastasis (TNM) Grouping Pattern Corresponding distribution of stages by TNM eighth edition and TNM ninth version for the whole series.

Table 2 summarizes the defining criteria and stage groupings of TNM-9 compared with TNM-8, and Figure 1 shows the pattern of T and N groupings with the corresponding distribution. With major changes in stage grouping, the distribution by TNM-9 became 17.3%, 40.4%, 38.0%, and 4.3% for stages I to IV, respectively.

**Table 2.** Classification Criteria and Stage Grouping by the American Joint Committee on Cancer (AJCC)/Union for International Cancer Control (UICC) Tumor-Node-Metastasis (TNM) System and Changes from the Eighth Edition to the Ninth Version

Stage	TNM eighth edition	TNM ninth version
**T category: no change**		
T1	Tumor confined to nasopharynx or extension to oropharynx and/or nasal cavity without parapharyngeal involvement	Tumor confined to nasopharynx or extension to any of the following without parapharyngeal involvement: (1) oropharynx; (2) nasal cavity (including nasal septum)
T2	Tumor with extension to parapharyngeal space and/or adjacent soft tissue involvement (medial pterygoid lateral pterygoid prevertebral muscles)	Tumor with extension to any of the following: (1) parapharyngeal space; (2) adjacent soft tissue involvement (medial pterygoid, lateral pterygoid, prevertebral muscles)
T3	Tumor with infiltration of bony structures at skull base cervical vertebra pterygoid structures and/or paranasal sinuses	Tumor with unequivocal infiltration into any of the following bony structures: (1) skull base (including pterygoid structures); (2) paranasal sinuses; (3) cervical vertebrae
T4	Tumor with intracranial extension, involvement of cranial nerves, hypopharynx, orbit, parotid gland, and/or extensive soft tissue infiltration beyond the lateral surface of the lateral pterygoid muscle	Tumor with any of the following extension/involvement: (1) intracranial extension; (2) unequivocal radiological and/or clinical involvement of cranial nerves; (3) hypopharynx; (4) orbit (including inferior orbital fissure); (5) parotid gland; (6) extensive soft tissue infiltration beyond the anterolateral surface of the lateral pterygoid muscle
**N category: addition of advanced extranodal extension as N3 criterion**		
N0	No regional lymph node metastasis	No tumor involvement of regional lymph node(s)
N1	Unilateral metastasis in cervical lymph node(s) and/or unilateral or bilateral metastasis in retropharyngeal lymph node(s), 6 cm or smaller in greatest dimension, above the caudal border of cricoid cartilage. Retropharyngeal (irrespective of laterality)	Tumor involvement of any of the following: (1) unilateral cervical lymph node(s); (2) unilateral or bilateral retropharyngeal lymph node(s). Tumor involvement in all of the following: (1) ≤6 cm in greatest dimension; (2) above the caudal border of cricoid cartilage; (3) without advanced extranodal extension
N2	Bilateral metastasis in cervical lymph node(s), 6 cm or smaller in greatest dimension above the caudal border of cricoid cartilage	Tumor involvement of bilateral cervical lymph nodes and all of the following: (1) ≤6 cm in greatest dimension; (2) above the caudal border of cricoid cartilage; (3) without advanced extranodal extension
N3	Unilateral or bilateral metastasis in cervical lymph node(s), larger than 6 cm in greatest dimension and/or extension below the caudal border of cricoid cartilage	Tumor involvement of unilateral or bilateral cervical lymph node(s) and any of the following: (1) >6 cm in greatest dimension; (2) extension below the caudal border of cricoid cartilage; (3) advanced radiologic extranodal extension with involvement of adjacent muscles, skin, and/or neurovascular bundle
**M category: subdivision of M1 into M1a and M1b**		
M0	No distant metastasis	No distant metastasis
M1	Distant metastasis	M1: distant metastasis; M1a: ≤3 metastatic lesions in ≥1 organs/sites; M1b: >3 metastatic lesions in ≥1 organs/sites
**Stage group: merging I and II; down-classifying III and IVA to II and III, respectively; exclusive inclusion of M1 into IV; subdivision of stages I and IV**		
I	T1, N0, M0	IA: T1-2, N0, M0; IB: T1-2, N1, M0
II:	T1, N1, M0; T2, N0-N1, M0	T1-2, N2, M0; T3, N0-2, M0
III	T1-2, N2, M0; T3, N0-2, M0	T4, any N, M0; any T, N3, M0
IV	IVA: T4 or N3 M0; IVB: any T, any N, M1	IVA: any T, any N, M1a; IVB: any T, any N, M1b

**Figure 2.**
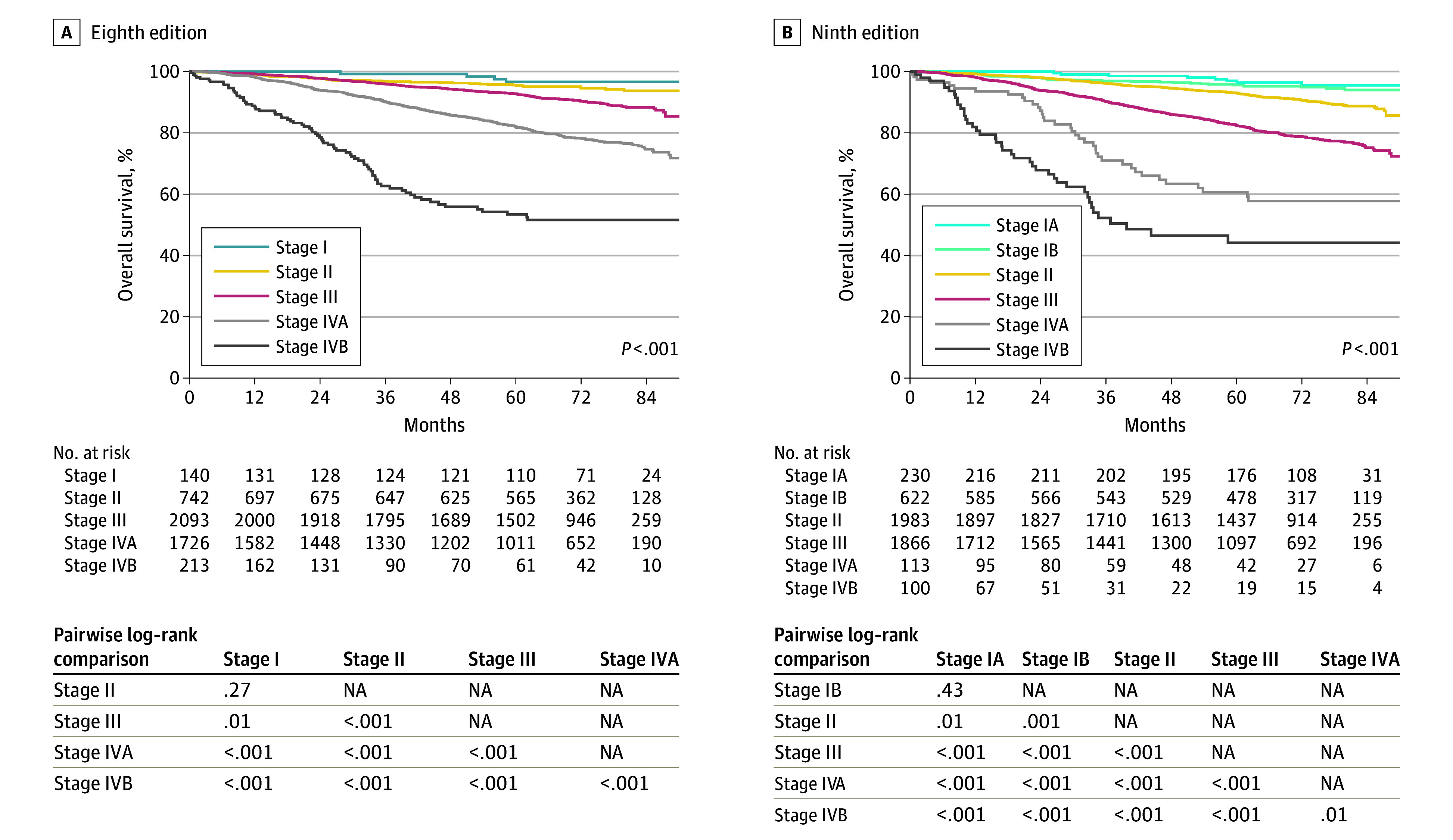
Prognostication on Overall Survival Stage of Tumor-Node-Metastasis (TNM) Eighth Edition and TNM Ninth Version for the Whole Series NA indicates not applicable.

Figure 2 shows the survival curves for different stages by the TNM-8 and TNM-9 systems, and eTable 3 in Supplement 1 summarizes the differences in OS and aHRs between the adjacent stages. The key drawback of TNM-8 was the lack of significant differences between stages I and II (5-year OS rates were 96.7% vs 95.6%; *P* = .27). With TNM-9, the distinction between stage I vs stage II became significant (5-year OS rates were 96.0% vs 93.1%; *P* < .001). Although there was no significant difference between IA and the more intensively treated IB subgroup (97.2% vs 95.5%; *P* = .47) due to preferential use of chemotherapy, the difference between IB vs II was significant (95.6% vs 93.1%; *P* = .001).

## Discussion

Contemporary patterns of care have been associated with substantially improved outcomes for NPC. This study showed that 89% of M0 patients received combined modality treatment, and the 5-year OS ranged narrowly from 97% to 82% for TNM-8 stage I to IVA (Figure 2). Even for M1 patients, most received intensive treatment, and 5-year OS reached 53%. Therefore, a reevaluation of the current TNM-8 system was needed.

Developing an optimal staging system is challenging. To ensure universal acceptance for global application, comprehensive considerations for clinical practicability, reproducibility, and relevance for treatment guidance are essential factors to consider, in addition to the fundamental demand for evidence-based statistical improvement. Staging is also a public health issue because there is increasing demand for data not only on overall incidence and mortality, but also stage distribution and the relative survival of different stages for formulating a strategic cancer control plan. The parsimony principle should be applied as far as possible, for which suggestions with marginal benefit ought to be deferred until further confirmation.

All the changes introduced in the upcoming TNM-9 for NPC were decided following stringent review processes as described previously. Table 2 summarized the final defining criteria and stage grouping for TNM-9 and the changes from TNM-8.

For the N category, advanced ENE will be added as an N3 criterion. There are various definitions for ENE, and interrater variation remains a practical issue. Thus, we only included unequivocal involvement of adjacent structures, which was a significant factor for OS, to avoid stage migration.^16,17^ This change in N category will ensure that patients with this adverse prognostic feature should be duly treated as locoregionally advanced disease.

Stage I will be expanded to include T1-2N0-1M0 subgroups. This will rectify the current lack of significant hazard distinction between stages I and II by TNM-8 (eTable 3 in Supplement 1). Following this change, TNM-8 stage III and IVA will be down-classified to TNM-9 stage II and III respectively, but the current categorization criteria (T3 or N2) as intermediate and (T4 or N3) poor will be maintained. All M0 patients will now be grouped into stages I to III, in line with many other solid cancers (particularly human papillomavirus [HPV]–mediated oropharyngeal cancer).^23^ One additional change is the subdivision of stage I into IA (T1-2N0M0) and IB (T1-2N1M0), because although the 2 subgroups had comparable OS, chemotherapy use was different, and IB patients had worse aHR (with adjustment for chemotherapy). Thus, it is prudent to have this separation to facilitate better treatment guidance and future outcomes analyses.

Stage IV is now used exclusively for M1 patients. Furthermore, to our knowledge, this is the first time in the staging of NPC that subdivision of the M category and corresponding stage IV was introduced, in line with HPV-positive oropharyngeal cancer. Although subcategorization of M1 had been advocated by several other studies,^24,25,26,27,28^ to our knowledge there is not yet a universally accepted classification. Our adoption of 3 or fewer vs more than 3 metastatic lesions, as the optimal cutoff was supported by the external validation study^7^ and other published studies.^25,28,29^ This may help in better selection of patients for intensive treatment rather than palliative treatment for all M1 patients (Figure 2).

There were 2 issues with discrepancies between our study and the external validation study,^7^ and these were duly considered by the AJCC and UICC panel (as described previously) before deciding not to include them into TNM-9. The first issue was the consideration of T category: our data suggested upclassifying medial ± lateral pterygoid muscle(s) involvement from T2 to T3, while the external validation study^7^ suggested down-classifying early skull base invasion from T3 to T2. Further studies for future consideration are encouraged, particularly as upclassifying pterygoid muscle involvement to T3 may provide a more logical anatomical transition instead of the current abrupt demarcation from T2 to T4.

The second issue is defining criteria for subdivision of the M1 category. The external validation study^7^ suggested including liver involvement in addition to the number of metastatic lesions. Although addition of the liver criterion may widen the distinction between subgroups (especially for patients who underwent PET-CT), the additional benefit was marginal, as only a few patients had 3 or fewer metastatic lesions with liver involvement. With the shift toward consideration of number of metastases for staging, the choice of imaging modality may play an important role in the accuracy of clinical stratification, and further evaluation is warranted.^7,28,30^

### Limitations

Despite the extensive work underpinning the TNM-9, there were notable limitations. First, this proposal was based on data from endemic regions where 97.5% of cases were nonkeratinizing carcinoma. Whether it is applicable to EBV-negative keratinizing tumors requires validation, but given that most NPC is the nonkeratinizing type, even in nonendemic regions,^18,31^ the proposed staging would be widely applicable. Second, while the staging scans were reviewed by experienced radiologists, interrater concordance was not formally assessed in our study (consistency was maximized through training and consensus building among the radiologists). Third, there was treatment heterogeneity pertaining to the use of chemotherapy among the participating institutions. To partially account for this, we performed separate MVA with and without chemotherapy as a covariate to assess the prognostic effect independently of treatment differences.

It may be critiqued that the proposed TNM-9 is still based solely on anatomic parameters; we agree that further refinement by incorporating nonanatomical factors is desirable. Plasma EBV-DNA is particularly valuable, but there are concerns that EBV-DNA testing is not universally available worldwide, and quantification methods are not harmonized, leading to marked variations in proposed cutoff values for risk stratification.^4,5,32,33,34,35^ Furthermore, not all patients with nonkeratinizing NPC (including those with advanced disease) have detectable circulating EBV-DNA.^36,37^ A pragmatic solution has yet to be implemented before this can become widely applicable.

## Conclusions

This diagnostic study represents the collaborative efforts by endemic centers, the China Anti-Cancer Association, and the AJCC/UICC committees to develop a fundamental TNM system for improving prognostication for NPC. The TNM-9 is superior to TNM-8 in major statistical aspects, including hazard discrimination, consistency, outcome prediction accuracy, and balanced distribution (eTable 5 in Supplement 1). The ninth version of the AJCC/UICC TNM staging will be launched for global application in January 2025 to provide a fundamental framework for clinicians in treatment decision-making, researchers in the design of future trials, and cancer registries for epidemiological studies. Building on this foundation, the working group will proceed to explore the feasibility of incorporating nonanatomical prognostic factors (particularly EBV-DNA) and work toward the ideal goal of precision prognostication with personalized risk stratification.

## Notes

Supplement 1. eTable 1. Patient characteristics of the whole series (N=4914) eTable 2. The significance of each anatomical structure within respective T-category in the non-metastatic Cohort (N=4701) eTable 3. Hazard Distinction on Overall Survival (OS) between adjacent stage-group by TNM-8 and TNM-9: in the non-metastatic Training Set, Validation Set and Whole Series eTable 4. Comparison of the non-metastatic Training Set versus Validation Set: patient characteristics, T- and N- Distribution eTable 5. Comparison of TNM-9 vs. TNM-8 in the non-metastatic Training Set, Validation Set and Bootstrap Replicates eTable 6. Significance of different features of distant metastases in the metastatic Cohort (N=213) eFigure 1. Stage-grouping in non-metastatic Training Set by recursive partitioning analysis with modification by adjusted hazard ratio eFigure 2. Prognostication of T1-2N0-1 subgroups in non-metastatic Training Set: overall survival, and distant failure-free survivalSupplement 2. Data sharing statement

Supplement 2. Data sharing statement
