# Supplementary material for: Ninth Version of the AJCC and UICC Nasopharyngeal Cancer TNM Staging Classification
Source: JAMA Oncol. 2024 Oct 10;10(12):1627–35. doi: 10.1001/jamaoncol.2024.4354 (PMC11581663; doi:10.1001/jamaoncol.2024.4354)
Supplement: Supplement 1. — eTable 1. Patient characteristics of the whole series (N=4914) eTable 2. The significance of each anatomical structure within respective T-category in the non-metastatic Cohort (N=4701) eTable 3. Hazard Distinction on Overall Survival (OS) between adjacent stage-group by TNM-8 and TNM-9: in the non-metastatic Training Set, Validation Set and Whole Series eTable 4. Comparison of the non-metastatic Training Set versus Validation Set: patient characteristics, T- and N- Distribution eTable 5. Comparison of TNM-9 vs. TNM-8 in the non-metastatic Training Set, Validation Set and Bootstrap Replicates eTable 6. Significance of different features of distant metastases in the metastatic Cohort (N=213) eFigure 1. Stage-grouping in non-metastatic Training Set by recursive partitioning analysis with modification by adjusted hazard ratio eFigure 2. Prognostication of T1-2N0-1 subgroups in non-metastatic Training Set: overall survival, and distant failure-free survivalSupplement 2. Data sharing statement [file jamaoncol-e244354-s001.pdf]

Supplemental Online Content

Pan J-J, Mai H-Q, Ng WT, et al. Ninth version of the AJCC and UICC nasopharyngeal cancer TNM staging classification. *JAMA Oncol*. Published online October 10, 2024. doi:10.1001/jamaoncol.2024.4354

eTable 1. Patient characteristics of the whole series (N=4914)
eTable 2. The significance of each anatomical structure within respective T-category in the non-metastatic Cohort (N=4701)
eTable 3. Hazard Distinction on Overall Survival (OS) between adjacent stage-group by TNM-8 and TNM-9: in the non-metastatic Training Set, Validation Set and Whole Series
eTable 4. Comparison of the non-metastatic Training Set versus Validation Set: patient characteristics, T- and N- Distribution
eTable 5. Comparison of TNM-9 vs. TNM-8 in the non-metastatic Training Set, Validation Set and Bootstrap Replicates
eTable 6. Significance of different features of distant metastases in the metastatic Cohort (N=213)
eFigure 1. Stage-grouping in non-metastatic Training Set by recursive partitioning analysis with modification by adjusted hazard ratio
eFigure 2. Prognostication of T1-2N0-1 subgroups in non-metastatic Training Set: overall survival, and distant failure-free survival

This supplemental material has been provided by the authors to give readers additional information about their work.

© 2024 Pan J-J et al. *JAMA Oncology.*

eTable 1. Patient characteristics of the whole series (N=4914)

	**Values**
**Whole series (N=4914) – Host factors**	
Age, mean (SD), y	48.1 (12.0)
Gender (Male)	3650 (74.3%)
Histology	
Keratinizing	98 (2.1%)
Non-keratinizing	4794 (97.5%)
Basaloid	22 (0.4%)
**Non-Metastatic Cohort (N=4701) – Investigation and Treatment**	
Staging investigation	
MRI	4701 (100%)
CT	111 (2.4%)
PET-CT	1493 (31.8%)
Treatment	
Radiotherapy	
Technique: IMRT/VMA/Tomotherapy	100%
Total dose, mean (SD), Gy	69.8 (1.6)
Dose/fraction, mean (SD), Gy	2.2 (0.3)
Chemotherapy	
None	530 (11.3%)
Concurrent	1055 (22.4%)
Concurrent+ Induction/Adjuvant	2427 (51.6%)
Induction/Adjuvant	689 (14.7%)
**Metastatic Cohort (N=213) – Metastatic features, Investigation and Treatment**	
Investigation for metastases	
Conventional scans	117 (54.9%)
PET-CT	96 (45.1%)
Number of Metastatic Lesions, Median (Range)	3 (1-30)
Number of Metastatic Sites, Median (Range)	1 (1-4)
Organ involved	
Lung	47 (22.1%)
Bone	144 (67.6%)
Liver	69 (32.4%)
Distant lymph node	34 (16.0%)
Treatment	
None	6 (2.8%)
Chemotherapy	199 (93.4%)
Radiotherapy for locoregional disease	171 (80.3%)
Surgery/radiotherapy for metastases	103 (48.4%)

MRI: magnetic resonance imaging; CT: computed tomography; PET: positron emission tomography; IMRT: intensity-modulated tomography, VMAT: volumetric arc therapy.

© 2024 Pan J-J et al. *JAMA Oncology.*

eTable 2. The significance of each anatomical structure within respective T-category in the non-metastatic Cohort (N=4701)

	**Anatomical structure**	**N**	**Unadjusted HR (95% CI, p-value)**	**Adjusted HR (without chemotherapy) (95% CI, p-value)**	**Adjusted HR (with chemotherapy) (95% CI, p-value)**
**Primary Tumor**					
T1	Gross tumor in nasopharynx	680	1	NA	NA
(N=722)	Nasal cavity ± Nasal Septum	28	0.57 (0.08-4.12, p=0.58)	NA	NA
	Oropharyngeal wall	22	1.50 (0.36-6.19, p=0.58)	NA	NA
**Adjacent soft tissues around nasopharynx**					
T2	Parapharyngeal ± Carotid space	773	1.67 (0.53-5.33, p=0.38)	NA	NA
(N=818)	Prevertebral Muscle	183	1.04 (0.62-1.77, p=0.87)	NA	NA
	Medial Pterygoid Muscle	83	2.77 (1.64-4.68, p<0.001)	2.52 (1.48-4.26, p=0.001)	2.59 (1.53-4.41, p<0.001)
	Lateral Pterygoid Muscle	4	7.38 (1.81-30.09, p=0.005) [a]	NA	NA
	Soft palate	7	0.05 (0.01-909.18, p=0.55)	NA	NA
**Skull base involvement**					
T3	Medial ± Lateral Pterygoid Plate	1031	1.20 (0.92-1.56, p=0.18)	NA	NA
(N=2097)	Pterygo-maxillary Fissure	152	1.29 (0.81-2.04, p=0.28)	NA	NA
	Pterygo-palatine Fossa	455	1.33 (0.99-1.80, p=0.06)	NA	NA
	Maxillary Antrum	114	0.99 (0.54-1.82, p=0.98)	NA	NA
	Ethmoid Sinus	84	0.87 (0.43-1.76, p=0.70)	NA	NA
	Sphenoid Sinus - Floor	1128	0.96 (0.74-1.25, p=0.76)	NA	NA
	Sphenoid Sinus - Other parts	177	1.35 (0.87-2.07, p=0.18)	NA	NA
	Clivus	1224	1.30 (0.99-1.71, p=0.06)	NA	NA
	Petrous Bone	729	1.12 (0.85-1.47, p=0.43)	NA	NA
	Petro-occipital Fissure	169	0.91 (0.54-1.53, p=0.71)	NA	NA
	Foramen Lacerum	530	1.11 (0.82-1.50, p=0.49)	NA	NA

© 2024 Pan J-J et al. *JAMA Oncology.*

	Foramen Ovale	136	0.94 (0.52-1.67, p=0.82)	NA	NA
	Foramen Rotundum	80	0.85 (0.38-1.91, p=0.69)	NA	NA
	Foramen Spinosum	63	1.20 (0.56-2.54, p=0.64)	NA	NA
	Carotid Canal	103	1.50 (0.87-2.58, p=0.14)	NA	NA
	Jugular Foramen	67	1.39 (0.71-2.71, p=0.33)	NA	NA
	Hypoglossal Canal	45	1.96 (1.01-3.82, p=0.048)	1.47 (0.75-2.87, p=0.27)	1.47 (0.75-2.89, p=0.26)
	Pituitary Fossa / Dorsum Sellae	17	1.04 (0.26-4.19, p=0.96)	NA	NA
	Cervical Vertebrae	24	1.30 (0.42-4.07, p=0.65)	NA	NA
**Remote structures**					
T4	Hypopharynx	8	0.81 (0.11-5.80, p=0.84)	NA	NA
(N=1064)	Infratemporal Fossa / Superficial Temporalis Muscle	64	1.47 (0.90-2.42, p=0.12)	NA	NA
	Parotid Gland	82	1.14 (0.70-1.88, p=0.60)	NA	NA
	Superior Orbital Fissure	37	1.74 (0.97-3.12, p=0.06)	NA	NA
	Inferior Orbital Fissure	104	1.95 (1.36-2.81, p<0.001)	1.72 (1.19-2.48, p=0.004)	1.68 (1.16-2.44, p=0.006)
	Orbit - Other Parts	38	1.74 (0.97-3.11, p=0.06)	NA	NA
	Cranial Nerve Involvement (clinical ± radiological)	381	0.90 (0.68-1.19, p=0.45)	NA	NA
	Cranial Nerve Involvement (radiological only)	227	0.56 (0.38-0.83, p=0.004)	0.77 (0.50-1.18, p=0.23)	0.81 (0.53-1.25, p=0.35)
	Cavernous Sinus	779	1.44 (1.03-2.01, p=0.03)	1.24 (0.86-1.77, p=0.25)	1.23 (0.86-1.77, p=0.26)
	Meckel's Cave	259	1.19 (0.88-1.61, p=0.27)	NA	NA
	Pituitary Gland / Suprasellar	38	0.77 (0.34-1.72, p=0.52)	NA	NA
	Meningeal / Cistern Infiltration	264	0.87 (0.64-1.20, p=0.41)	NA	NA
	Cerebral Lobes	26	0.48 (0.15-1.53, p=0.22)	NA	NA

Multivariable analyses: covariables include age, gender, N-category, significant primary tumor features ± chemotherapy.

HR: hazard ratio; CI: confidence interval;

© 2024 Pan J-J et al. *JAMA Oncology.*

[a]Not applicable for multivariable analysis due to small sample size.

© 2024 Pan J-J et al. *JAMA Oncology.*

eTable 3. Hazard Distinction on Overall Survival (OS) between adjacent stage-group by TNM-8 and TNM-9: in the non-metastatic Training Set, Validation Set and Whole Series

	TNM-9				TNM-8			
**Stage**	**Group**	**5-year OS**	**AHR without chemotherapy (95%CI), p value**	**AHR with chemotherapy (95%CI), p value**	**Group**	**5-year OS**	**AHR without chemotherapy (95%CI), p value**	**AHR with chemotherapy (95%CI), p value**
**Training Set (N=3205)**								
IB vs IA	T1-2N1 vs T1-2N0	95.5 vs 97.2	2.09 (0.83-5.27, p=0.12)	2.84 (1.05-7.71, p=0.04)	NA	NA	NA	NA
II vs IB	T3/N2 vs T1-2N1	92.8 vs 95.5	1.71 (1.09-2.70, p=0.02)	1.83 (1.15-2.89, p=0.01)	NA	NA	NA	NA
II vs I	T3/N2 vs T1-2N0-1	92.8 vs 95.9	1.97 (1.31-2.97, p=0.001)	2.30 (1.50-3.53, p<0·001)	T2/N1 vs T1N0	95.4 vs 96.3	2.28 (0.69-7.57, p=0.18)	2.51 (0.73-8.61, p=0.14)
III vs II	T4/N3 vs T3/N2	81.2 vs 92.8	2.53 (2.04-3.13, p<0·001)	2.62 (2.11-3.24, p<0·001)	T3/N2 vs T2/N1	92.4 vs 95.4	1.75 (1.16-2.62, p=0.007)	1.95 (1.29-2.95, p=0.002)
IVA vs III	NA	NA	NA	NA	T4/N3 vs T3/N2	80.8 vs 92.4	2.47 (2.01-3.05, p<0·001)	2.55 (2.07-3.15, p<0·001)
**Validation Set (N=1496)**								
IB vs IA	T1-2N1 vs T1-2N0	96.0 vs 96.5	1.37 (0.29-6.48, p=0.69)	1.57 (0.29-8.49, p=0.60)	NA	NA	NA	NA
II vs IB	T3/N2 vs T1-2N1	93.7 vs 96.0	1.97 (0.93-4.18, p=0.08)	1.89 (0.89-4.03, p=0.10)	NA	NA	NA	NA
II vs I	T3/N2 vs T1-2N0-1	93.7 vs 96.1	2·20 (1·10-4·37, p=0·03)	2.05 (1.01-4.15, p=0.045)	T2/N1 vs T1N0	96.0 vs 97.4	1.81 (0.23-14.31, p=0.58)	2.02 (0.24-16.97, p=0.52)
III vs II	T4/N3 vs T3/N2	85.1 vs 93.7	2.32 (1.63-3.29, p<0·001)	2.33 (1.64-3.32, p<0·001)	T3/N2 vs T2/N1	93.5 vs 96.0	2.11 (1.03-4.29, p=0.04)	2.06 (1.01-4.24, p=0.049)
IVA vs III	NA	NA	NA	NA	T4/N3 vs T3/N2	84.6 vs 93.5	2.29 (1.62-3.23, p<0·001)	2.30 (1.63-3.25, p<0·001)
**Whole Series (4914)**								
IB vs IA	T1-2N1 vs T1-2N0	95.6 vs 97.0	1.78 (0.81-3.93, p=0.15)	2.46 (1.04-5.85, p=0.04)	NA	NA	NA	NA
II vs IB	T3/N2 vs T1-2N1	93.1 vs 95.6	1.83 (1.24-2.69, p=0.002)	1.90 (1.28-2.80, p=0.001)	NA	NA	NA	NA

© 2024 Pan J-J et al. *JAMA Oncology.*

II vs I	T3/N2 vs T1-2N0-1	93.1 vs 96.0	2.07 (1.46-2.95, p<0·001)	2.29 (1.60-3.30, p<0·001)	T2/N1 vs T1N0	95.6 vs 96.7	2.14 (0.76-6.03, p=0.15)	2.52 (0.86-7.33, p=0.09)
III vs II	T4/N3 vs T3/N2	82.5 vs 93.1	2.46 (2.05-2.95, p<0·001)	2.52 (2.10-3.03, p<0·001)	T3/N2 vs T2/N1	92.8 vs 95.6	1.87 (1.32-2.66, p<0·001)	2.02 (1.41-2.89, p<0·001)
IVA vs III	M1a vs T4/N3	60.7 vs 85.2	2.07 (1.47-2.91, p<0·001)	2.08 (1.48-2.93, p<0·001)	T4/N3 vs T3/N2	82.0 vs 92.8	2.41 (2.02-2.89, p<0·001)	2.47 (2.06-2.96, p<0·001)
IVB vs IVA	M1b vs M1a	44.2 vs 60.7	1.86 (1.18-2.91, p=0·007)	1.92 (1.22-3.03, p=0.005)	M1 vs T4/N3	53.4 vs 82.0	2.74 (2.13-3.51, p<0·001)	2.80 (2.18-3.59, p<0·001)

Multivariable analyses: covariables include age, gender ± chemotherapy.

© 2024 Pan J-J et al. *JAMA Oncology.*

eTable 4. Comparison of the non-metastatic Training Set versus Validation Set: patient characteristics, T- and N- Distribution

	**Training Set (n=3205)**	**Validation Set (n=1496)**	**p-value**
**Comparison of basic patient characteristics, T- and N-category distribution**			
Age, mean (SD), y	48.1 (12.1)	47.7 (11.7)	0.39
Gender (Male%)	2354 (73.4%)	1121 (74.9%)	0.28
T-category by TNM-8			
T1	498 (15.5%)	224 (15.0%)	0.91
T2	559 (17.4%)	259 (17.3%)	
T3	1419 (44.3%)	678 (45.3%)	
T4	729 (22.7%)	335 (22.4%)	
N-category by TNM-8			
N0	374 (11.7%)	177 (11.8%)	0.99
N1	1281 (40.0%)	602 (40.2%)	
N2	986 (30.8%)	455 (30.4%)	
N3	564 (17.6%)	262 (17.5%)	
N-category by TNM-9			
N0	374 (11.7%)	177 (11.8%)	0.99
N1	1226 (38.3%)	576 (38.5%)	
N2	910 (28.4%)	417 (27.9%)	
N3	695 (21.7%)	326 (21.8%)	

© 2024 Pan J-J et al. *JAMA Oncology.*

eTable 5. Comparison of TNM-9 vs. TNM-8 in the non-metastatic Training Set, Validation Set and Bootstrap Replicates

Statistical Aspects	Statistical Test	Training Set		Validation Set		Bootstrap Validation			
		TNM-8	TNM-9	TNM-8	TNM-9	TNM-8	TNM-9	Improvement	p
Balance	Distribution balance	0.614	0.294	0.647	0.326	0.625 (0.620-0.629)	0.304 (0.304-0.304)	0.321 (0.316-0.325)	<0.001
Consistency	Hazard consistency	2.725	2.160	0.988	1.112	4.326 (2.118-7.054)	3.914 (2.219-6.189)	0.380 (-1.065-1.996)	<0.001
Discrimination	Hazard Discrimination	0.868	0.693	0.009	0.003	1.234 (0.046-5.673)	0.689 (0.002-7.026)	0.222 (-2.482-2.921)	<0.001
Outcome Prediction	C-Index	0.718	0.722	0.705	0.705	0.716 (0.693-0.737)	0.718 (0.696-0.740)	0.003 (-0.003-0.010)	<0.001
	Likelihood Difference	43.009	66.828	16.208	23.015	59.142 (43.353-76.638)	89.094 (65.600-115.944)	29.945 (19.670-42.123)	<0.001
Accuracy	Brier Score	0.089	0.089	0.078	0.078	0.095 (0.088-0.103)	0.095 (0.087-0.103)	0 (0-0.001)	<0.001
Overall rank		2	1	2	1	2	1[a]	NA	NA

[a]The Ninth Version (TNM-9) ranked 1st as compared to the Current Eighth Edition (TNM-8) ranked 2nd in 4595/5000 (92%) of the replicates

© 2024 Pan J-J et al. *JAMA Oncology.*

eTable 6. Significance of different features of distant metastases in the metastatic Cohort (N=213)

		**N**	**Unadjusted HR (95% CI, p-value)**	**Adjusted HR (95% CI, p-value)** [a]
No. of lesions	≤3 vs >3	113 vs 100	1.77 (1.13-2.77, p=0.01)	1.76 (1.11, 2.77, p=0.02)
No. of sites	≤2 vs >2	196 vs 17	2.53 (1.30-4.93, p=0.006)	1.96 (0.99, 3.87, p=0.052)
Organ involved	Lung	24	1	NA
	Bone	93	2.25 (0.87-5.82, p=0.09)	NA
	Liver	26	2.48 (0.85-7.28, p=0.10)	NA
	Distant node	8	3.55 (0.95-13.25, p=0.06)	NA

Multivariable analyses: covariables include age, gender, and significant metastatic features on univariable analyses

[a]Among the 151 patients with single organ involvement

HR: hazard ratio; CI: confidence interval.

© 2024 Pan J-J et al. *JAMA Oncology.*

eFigure 1. Stage-grouping in non-metastatic Training Set by recursive partitioning analysis with modification by adjusted hazard ratio

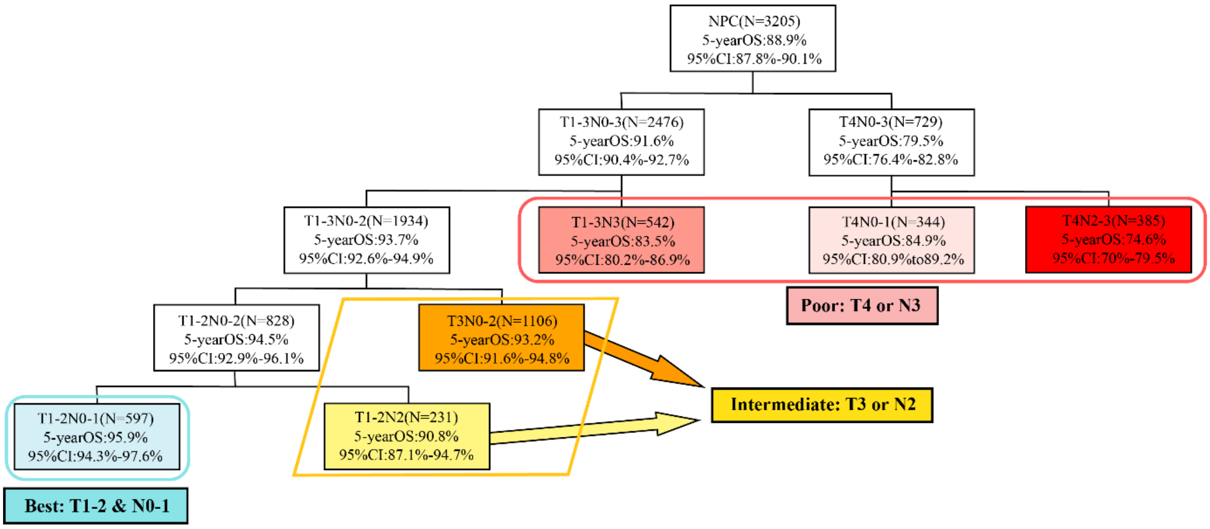

© 2024 Pan J-J et al. *JAMA Oncology.*

eFigure 2. Prognostication of T1-2N0-1 subgroups in non-metastatic Training Set: overall survival, and distant failure-free survival

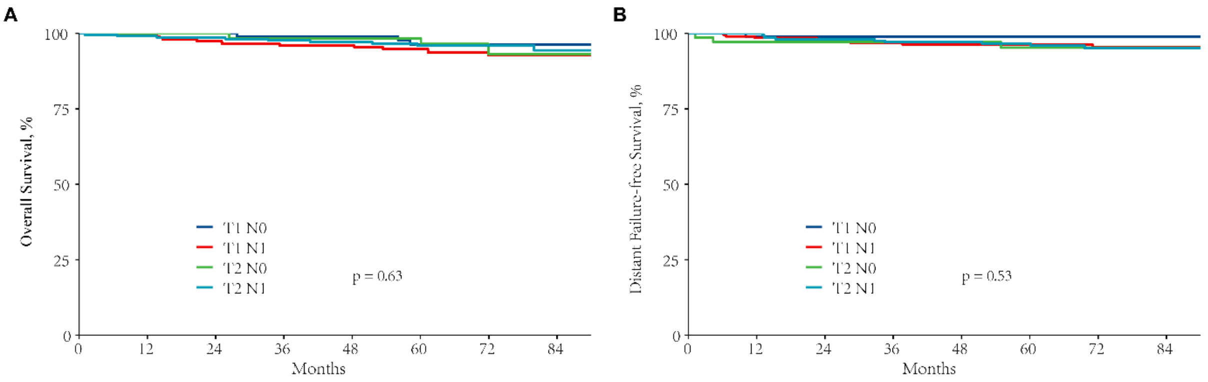

A — No. at risk

T1 N0	97	91	88	86	83	75	51	18
T1 N1	211	198	190	181	176	159	114	48
T2 N0	68	66	64	59	57	50	28	5
T2 N1	221	210	204	195	189	174	110	34

B — No. at risk

T1 N0	97	91	88	86	83	75	51	18
T1 N1	211	194	186	176	170	154	110	48
T2 N0	68	64	62	58	56	47	27	5
T2 N1	221	209	202	192	186	170	106	32
