# Supplementary material for: Ninth Version of the AJCC and UICC Nasopharyngeal Cancer TNM Staging Classification
Source: JAMA Oncol. 2024 Oct 10;10(12):1627–35. doi: 10.1001/jamaoncol.2024.4354 (PMC11581663; doi:10.1001/jamaoncol.2024.4354)
Supplement: Supplement 2. — Data sharing statement [file jamaoncol-e244354-s002.pdf]

Data Sharing Statement

Pan. Ninth Version of the AJCC and UICC Nasopharyngeal Cancer TNM Staging Classification. *JAMA Oncol*. Published October 10, 2024. doi:10.1001/jamaoncol.2024.4354

Data
Data available: Yes
Data types: Data dictionary
How to access data: The request for data must be sent to corresponding author at awmlee@hku.hk
When available: With publication

Supporting Documents
Document types: None

Additional Information
Who can access the data: Data will be shared after application to the governance panel, successful approval of a proposal, with a signed data access agreement.
Types of analyses: Regarding the types of analyses for the data dictionary will be made available, we are specifically interested in conducting a well-designed external validation of TNM Staging of Nasopharyngeal Cancer 9th version.
Mechanisms of data availability: Data will be shared after application to the governance panel, successful approval of a proposal, with a signed data access agreement.
